# Curcumin Relieves Chronic Unpredictable Mild Stress-Induced Depression-Like Behavior through the PGC-1*α*/FNDC5/BDNF Pathway

**DOI:** 10.1155/2021/2630445

**Published:** 2021-12-14

**Authors:** Yanqin Wu, Fusheng Sun, Yujin Guo, Yumao Zhang, Li Li, Ruili Dang, Pei Jiang

**Affiliations:** ^1^Department of Pharmacy, The Eighth Affiliated Hospital of Sun Yat-Sen University, Shenzhen, 518000 Guangdong, China; ^2^Department of Pharmacy, Qingdao Municipal Hospital, Qingdao, 266005 Shandong, China; ^3^Institute of Clinical Pharmacy, Jining First People's Hospital, Jining Medical University, Jining, 272000 Shandong, China

## Abstract

**Methods:**

All rats were randomly divided into four groups, namely, control, CUMS, CUMS + CUR, and CUMS + CUR + SR18292 (PGC-1*α* inhibitor). Behavioral tests were conducted to assess the antidepressant-like effects of CUR. The expressions of PGC-1*α*, estrogen-related receptor alpha (ERR*α*), FNDC5, and BDNF were determined to investigate the regulatory effects of CUR on the PGC-1*α*/FNDC5/BDNF pathway. The PGC-1*α* inhibitor SR18292 was used to explore the role of PGC-1*α* in the induction of BDNF by CUR.

**Results:**

Daily gavage of 100 mg/kg CUR successfully attenuated the abnormal behaviors induced by CUMS and effectively prevented CUMS-induced reduction of PGC-1*α*, ERR*α*, FNDC5, and BDNF expressions. CUR also enhanced PGC-1*α* and ERR*α* translocation from cytoplasm to nucleus. Furthermore, we found that CUR supplementation effectively promoted neurocyte proliferation and suppressed neuronal apoptosis induced by CUMS. Of note, the PGC-1*α* inhibitor SR18292 remarkably reversed the beneficial effects of CUR on depressed rats, indicating an important role of PGC-1*α* in the antidepressant-like effects of CUR.

**Conclusion:**

Collectively, our data evaluating the neuroprotective action of CUR in the CUMS rats highlights the involvement of the PGC-1*α*/FNDC5/BDNF pathway in the antidepressant-like effects of CUR.

## 1. Introduction

Depression is the most frequent psychiatric disorder, with more than 264 million people affected worldwide [1]. Depression is associated with extreme suffering for individuals and increased risk of suicide, and it inflicts an enormous economic burden on society. Multiple lines of evidence indicate that neuroinflammation, oxidative stress, mitochondrial dysfunction, and decreased monoamine neurotransmitters and neurotrophic factors are involved in the development of depression [2]. As a member of the neurotrophin family, brain-derived neurotrophic factor (BDNF) is one of the best-studied neurotrophic factors. It is widely expressed in the central nervous system and serves essential functions in synapse formation, synaptic plasticity, and neuronal maturation in the brain. Studies have revealed BDNF's involvement in processes of learning, memory, and cognition, as well as in mood-related behaviors [3]. In the past two decades, BDNF has been widely studied in the context of neuropsychiatric disorders especially for depressive disorders, bipolar disorder, schizophrenia, addiction, and eating disorders [4]. Accumulating evidence suggests that BDNF could play an important role in the treatment of depression [4, 5]; however, the biochemistry has not been defined. In particular, the intracellular signaling pathways necessary for BDNF's antidepressant-like effects remain elusive.

Peroxisome proliferator-activated receptor *γ* coactivator 1*α* (PGC-1*α*) was initially discovered as a transcriptional coactivator [6]. It is known as a metabolic regulator which plays a critical role in the maintenance of glucose, lipid, and energy homeostasis [7]. Subsequent work has demonstrated the importance of PGC-1*α* in the inhibition of neurodegeneration [8]. Studies have suggested that PGC-1*α* improves learning and memory through regulation of the downstream membrane protein, fibronectin type III domain-containing 5 (FNDC5) [9–11]. FNDC5 is identified as a PGC-1*α*-dependent myokine, which is known to be profoundly expressed in the brain in many regions, including the hypothalamus, as well as the hippocampus [12]. A previous study conducted by Wrann et al. [10] showed a strong correlation between PGC-1a and FNDC5 gene expression and demonstrated that PGC-1a is a regulator of neuronal FNDC5 gene expression in the brain. More researchers subsequently have reported that FNDC5 can modulate BDNF expression and release in the hippocampus [8, 11, 13]. Considering the role of BDNF in the inhibition of depression, PGC1a/FNDC5/BDNF has been determined to be a critical pathway for neuroprotection; as such, it is expected to be an effective target for therapeutic interventions in depressive disorders [14].

Curcumin (CUR), the major active component extracted from the Chinese medicine *Curcuma longa*, has been reported to possess neuroprotective effects [14]. CUR's potential antidepressant-like effects have been highlighted in many preclinical trials conducted on rats and mice models of depression [15–18]. Several clinical trials have also been conducted on the potential effectiveness of CUR in depression; however, they have yielded conflicting conclusions [19–22]. Nevertheless, a meta-analysis reviewing ten clinical trials concludes that CUR might be effective as adjunctive treatment in depressive disorders, indicating the promising efficacy of CUR in depression [19–22]. More and more studies have discussed the potential mechanism of CUR's antidepressant-like effects. Our previous study [15] found that CUR could restore changes in proinflammatory cytokines and the indolamine-2, 3-dioxygenase- (IDO-) kynurenine pathway in the hippocampus of CUMS rats, which might ultimately contribute to its antidepressant-like effect. Another study conducted by our teammates Liao et al. [16] suggested that the possible antidepressant-like effects of CUR are associated with oxidative stress and with changes in the activation of erythroid-2-related factor 2 (Nrf2) in the brain. Moreover, Liao et al. [16] found that CUR could reverse the decreased expression of BDNF. Other studies have also indicated CUR's ability to increase BDNF levels [23–25]. These studies point strongly to an association between the antidepressant-like effects of CUR and the regulation of BDNF levels; however, no studies so far have explored the detailed mechanisms of this association.

Therefore, in our present study, we used a chronic unpredictable mild stress- (CUMS-) induced depression model to investigate whether the antidepressant-like effects of CUR are associated with the activation of the PGC-1*α*/FNDC5/BDNF pathway.

## 2. Methods

### 2.1. Animals

Sprague Dawley (SD) rats (male, weight: 180-220 g, age: 40-45 days; Beijing Vitonlihua Experimental Animal Technology Co. Ltd, Beijing, China) were initially housed in groups in a temperature-controlled environment under a 12/12 h light/dark cycle. Food and water were freely available during the whole experiment except for rats specifically kept under deprivation conditions. This study was approved by the Animal Care and Use Committee of the Eighth Affiliated Hospital of Sun Yat-sen University. All experiments were performed according to the Guide for Care and Use of Laboratory Animals (Chinese Council).

### 2.2. Treatments

Rats were randomly assigned to four groups (*n* = 7): control, CUMS, CUMS + CUR, CUMS + CUR + SR18292 (PGC-1*α* inhibitor). The trial was conducted over 6 weeks. The control group received no stress treatment (CUMS) and no drugs. The other three groups were exposed to a stress protocol (CUMS) every day for 6 weeks. In addition, the CUR groups received daily gavage of 100 mg/kg CUR (suspended in 0.5% Tween 80, purchased from Sigma Chemical Co., USA) 60 min prior to CUMS. The CUMS + CUR + SR18292 rats received SR18292 (dissolved in DMSO, purchased from Macklin, Shanghai, China) via intraperitoneal injection at a dose of 40 mg/kg every day in the last week for a total of seven injections. The doses of CUR and SR18292 were based on previous studies [15, 16].

**Figure 1:**
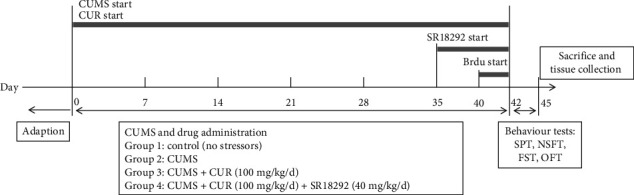
Timeline of experimental procedures. CUMS: chronic unpredictable mild stress; CUR: curcumin; SPT: sucrose preference test; NSFT: novelty-suppressed feeding test; OFT: open-field test; FST: forced swimming test.

At the end of six weeks, behavioral tests were carried out. Then, all the rats were sacrificed under anesthesia with an intraperitoneal injection of 1% sodium pentobarbital (50 mg/kg). Blood was collected, and the brains were rapidly removed after cardiac perfusion with phosphate-buffered saline (PBS) (pH = 7.2). The hippocampus samples were dissected on a cold surface and thoroughly washed with cold physiological saline and then stored at −80°C until analysis. The timeline of the experimental procedures is shown in Figure 1.

### 2.3. CUMS Procedure

Rats in the control group were housed in groups of 3-4 per cage in a separate room while rats in the CUMS groups were housed individually and received random unpredictable stress for 6 consecutive weeks. The CMS procedure was performed as previously described with minor modification [26]. Stress stimuli included cage tilting for 24 h; damp bedding for 24 h; fasting for 24 h; water deprivation for 24 h, with an empty bottle provided during the last hour; light–dark-cycle reversal (12 h/12 h), behavior restriction for 2 h; 30 min noise, 5 min tail pinch. Rats received one of these stressors continuously, individually, and randomly for six weeks. The same stressor was not applied in 2 consecutive days.

### 2.4. Behavioral Tests

#### 2.4.1. Sucrose Preference Test (SPT)

The procedure was performed as described previously [27] with minor modifications. Before the SPT test, all the rats were housed individually and provided two bottles containing 1% sucrose solution for 48 h to habituate them to the taste of sucrose. After 14 h of water deprivation, two preweighed bottles—one containing 1% sucrose solution and another containing tap water—were given to each rat. After 1 h, the bottles were weighed again, and the weight difference in each bottle was considered the intake of each rat. The sucrose preference was calculated as a percentage of the consumed sucrose solution relative to the total amount of liquid intake.

#### 2.4.2. Open-Field Test (OFT)

The OFT was performed as described previously [28]. The test was performed in a square arena consisting of a 76 × 76 cm gray wooden box with 42 cm high boundary walls and with the floor divided into 25 equal squares by black lines. Each rat was placed into the center of the open field and allowed to move freely for 5 min. The apparatus was cleaned with ethanol and water prior to each test session to remove olfactory cues. The number of crossing and rearing was recorded by the observer blind to the treatment condition of the animal to assess locomotor activity and exploratory behavior.

#### 2.4.3. Forced Swimming Test (FST)

The FST was conducted according to a classic paradigm [29] with slight modification. Before the FST test, each rat was placed in a plastic drum (45 cm height, 25 cm diameter) containing approximately 35 cm of water (24 ± 1°C) for a 15 min pretest. After swimming, rats were dried with towels and placed back in their home cage. After 24 h, the rats were exposed to the same experimental conditions outlined above for a 5 min FST. We changed the water before each trial to remove olfactory disturbance. Immobility time was scored by an experienced observer blind to the experiment design and was defined as floating passively and only making slight movements to keep the head above water.

#### 2.4.4. Novelty-Suppressed Feeding Test (NSFT)

The NSFT was performed according to a previous method [30]. Before NSFT, all the rats were deprived of food for 24 h in their home cages. A small amount of food was placed on a piece of white paper (10 × 10 cm) in the center of an open field (75 × 75 × 40 cm). The rats were allowed to explore the open field freely for 8 min. The latency time was recorded in our study, defined as the time it took for each rat to approach and take the first bite of the food. Immediately afterward, the animals were transferred to their home cages and were provided the same amount of food as in the open field. Total food intake for the next 5 min in each cage was weighed to avoid the influence of the animals' appetite.

### 2.5. Western Blotting Analysis

Western blotting analysis was conducted according to the previous method [31]. Total protein from the hippocampus was prepared, and the Bradford method was used to determine the protein concentration. Samples were loaded on precast 12% SDS-PAGE gels with 10 *μ*g of protein in each lane. Then, the proteins in the gels were transferred to a PVDF membrane and blocked in 5% nonfat dry milk in TBS-T for 1 h (25 mM Tris, 150 mM NaCl, pH 7.5, 0.05% Tween-20). The following antibodies and concentrations were used overnight at a temperature of 4°C: PGC-1*α* (ab106814, 1 : 1000, Abcam), estrogen-related receptor alpha (ERR*α*, #13826, 1 : 1000, Cell Signaling Technology), FNDC5 (23995-1-AP, 1 : 1000; Proteintech), BDNF (28205-1-AP 1 : 1000; Proteintech), and *β*-actin (8046S, 1 : 1000, Cell Signaling Technology). The membrane was then probed for 40 min with an HRP conjugated secondary antibody. Finally, the film signal was digitally scanned and quantified using Image-Pro Plus 6.0. The values were normalized to *β*-actin as internal standard.

### 2.6. Quantitative Real-Time PCR (qPCR)

Total RNA from the hippocampal homogenates was isolated using Trizol reagent (Invitrogen, USA) following the manufacturer's instructions. The mRNA expressions of PGC-1*α*, ERR*α*, FNDC5, BDNF, Bax, Bcl-xl, and Bcl-2 were detected. qPCR was performed on a Bio-Rad Cx96 Detection System (Biorad, USA) with an SYBR green PCR kit (Applied Biosystems, USA) and gene-specific primers. 5 ng cDNA samples were used, and thermocycling conditions were as follows: initial denaturation at 95°C for 2 min, followed by 40 cycles of amplification at 95°C for 15 sec and 60°C for 30 sec. Each cDNA was tested in triplicate. Relative quantitations for PCR products were normalized to *β*-actin as internal standard. The oligonucleotide primers used in the qPCR analysis are listed in Table 1.

### 2.7. Nissl Staining

Nissl staining was performed as previously described [32]. Brain sections (4 *μ*m thickness) were deparaffinized with xylene, rehydrated in a graded series of alcohol, and finally treated with Nissl staining solution for 5 min. Damaged neurons were shrunken or contained vacuoles, while the normal neurons had relatively large and full cell bodies with round nuclei. We calculate the average intensities or cell counts in the same sections from seven rats per group with Image-Pro Plus 7.0. Investigators were blinded to the experimental groups.

### 2.8. Immunofluorescence Analysis

The immunofluorescence analysis was performed according to the previous study [33]. The paraffin tissue blocks were cut in sections using a microtome. After washing three times with PBS, the brain sections were blocked in PBS at 37°C for 2 h using 10% goat serum (Solarbio, China) and 0.3% Triton X-100 (Solarbio, China). Then, the sections were incubated with primary antibodies (PGC-1*α*, ab106814, 1 : 300, Abcam) overnight at 4°C. After washing with PBS, the sections were incubated with secondary antibodies (4412, 1 : 1000, Cell Signaling Technology) for 1 h and with 4′,6-diamidino-2-phenylindole (Solarbio, China) for 5 min. Fluorescence was observed with a fluorescence microscope. The results were analyzed using Image-Pro Plus software. Investigators were blinded to the experimental groups.

### 2.9. Bromodeoxyuridine (Brdu) Treatment

The Brdu treatment was conducted according to the previous study [34]. BrdU (100 mg/kg) was injected intraperitoneally once daily for 3 consecutive days before the brain slice collection. After washing in 0.1 M borate buffer (pH = 8.5) for 30 min, the thirty-*μ*m-thick coronal sections containing dentate gyrus (DG) were collected and pretreated with hydrochloric acid at 37°C for 30 min and then incubated with 3% BSA for 1 h. Then, the sections were incubated with the antibody for BrdU, followed by Alexa Fluor secondary antibody. Photomicrographs were obtained with a FluoView FV1000 microscope.

### 2.10. Statistical Analysis

All statistical procedures were performed using Statistical Package for the Social Sciences (SPSS) 24.0. Data were expressed as mean ± SD. All the data were analyzed statistically by one-way analysis of variance (ANOVA) with Tukey post hoc multiple comparisons. *p* < 0.05 was considered as statistically significant.

## 3. Results

### 3.1. Effects of CUR on Behavioral Tests

**Figure 2:**
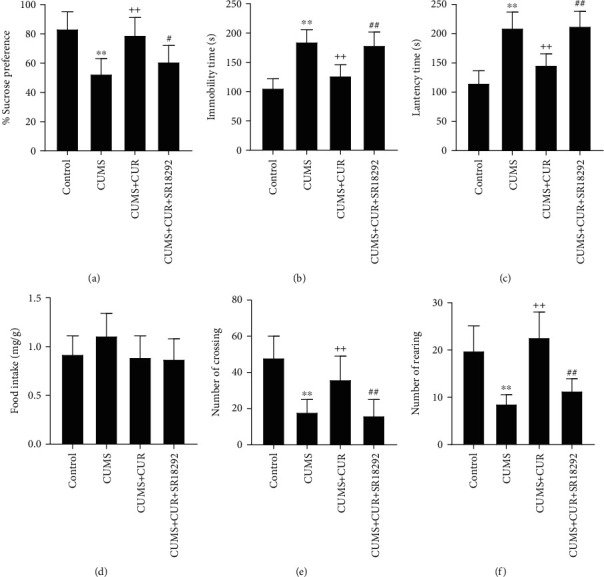
(a) Sucrose preference in SPT. (b) Immobility time in FST. (c) Latency time in NSFT. (d) Food intake in NSFT. (e) Number of crossing and (f) number of rearing in OPT. Data are expressed as means ± SD (*n* = 7). ^∗^*p* < 0.05 and ^∗∗^*p* < 0.01 compared to the control group. ^+^*p* < 0.05 and ^++^*p* < 0.01 compared to the CUMS group. ^#^*p* < 0.05 and ^##^*p* < 0.01 compared to the CUMS + CUR group.

The results of behavioral tests are shown in Figures 2(a)–2(f). After stress exposure for six weeks, the CUMS rats represented depression-like state with reduced source preference in SPT (*p* value < 0.01), prolonged immobility (*p* value < 0.01) in FST, and increased latency time (*p* value < 0.01) in NSFT compared to the rats in the control group. In addition, the CUMS rats displayed a reduction of the number of crossing (*p* value < 0.01) and rearing (*p* value < 0.01) in OPT.

In comparison, the CUMS + CUR rats showed significantly increased sucrose preference (*p* value < 0.01), decreased immobility time (*p* value < 0.01) and latency time (*p* value < 0.01), and increased number of crossings (*p* value < 0.01) and rearings (*p* value < 0.01).

When compared to CUMS + CUR group, in the CUMS + CUR + SR18292 group, the administration of PGC-1 inhibitor SR18289 successfully decreased sucrose preference (*p* value < 0.05), increased immobility time (*p* value < 0.01) and latency time (*p* value < 0.01), and decreased the number of crossings (*p* value < 0.01) and rearings (*p* value < 0.01).

No significant differences in food intake were observed in NSFT (Figure 2(d)), which is consistent with the results of the previous studies.

### 3.2. Effects of CUR on PGC-1 and ERR*α* Expressions

**Figure 3:**
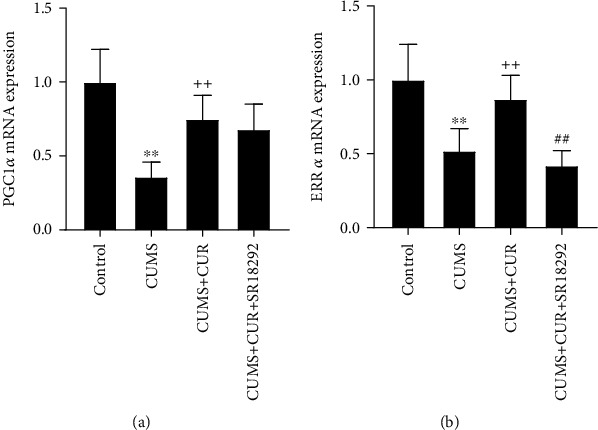
(a) and (b) Relative mRNA expressions of PGC-1 and ERR*α*. Data are expressed as means ± SD (*n* = 7). ^∗^*p* < 0.05 and ^∗∗^*p* < 0.01 compared to the control group. ^+^*p* < 0.05 and ^++^*p* < 0.01 compared to the CUMS group. ^#^*p* < 0.05 and ^##^*p* < 0.01 compared to the CUMS + CUR group.

As shown in Figures 3(a) and 3(b), the mRNA levels of PGC-1 (*p* value < 0.01) and ERR*α* (*p* value < 0.01) were significantly decreased in the CUMS group compared to the control group, and CUR treatment significantly increased the mRNA expressions of PGC-1 (*p* value < 0.01) and ERR*α* (*p* value < 0.01) in the CUMS + CUR group compared to the CUMS group. The CUMS + CUR + SR18292 group showed a significant decrease in the mRNA levels of PGC-1 (*p* value < 0.01) and a slight but not significant decrease in the mRNA levels of ERR*α* when compared to the CUMS + CUR group.

**Figure 4:**
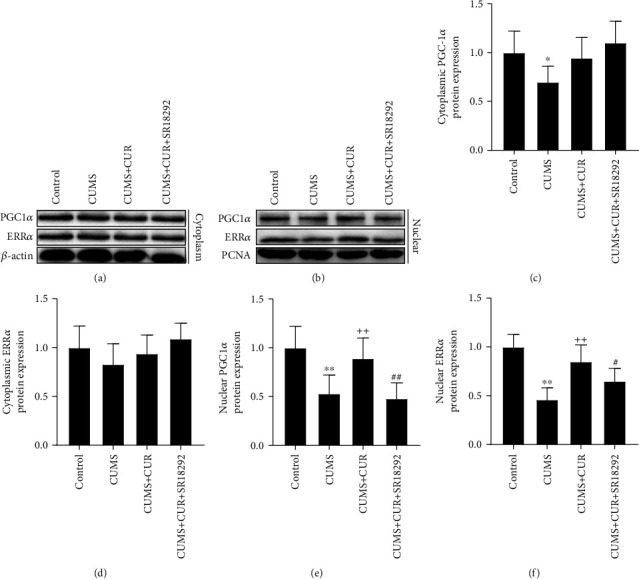
(a) and (b) Western blot bands indicating the protein expressions of PGC-1*α* and ERR*α* in the hippocampus. (c)–(f) Protein expressions of PGC-1*α* and ERR*α* normalized to that of the *β*-actin internal control. Data are expressed as means ± SD (*n* = 7). ^∗^*p* < 0.05 and ^∗∗^*p* < 0.01 compared to the control group. ^+^*p* < 0.05 and ^++^*p* < 0.01 compared to the CUMS group. ^#^*p* < 0.05 and ^##^*p* < 0.01 compared to the CUMS + CUR group.

As shown in Figures 4(a)–4(d), the rats in the four groups represented no significant changes in the cytoplasmic protein expressions of PGC-1 and ERR*α*, except that the cytoplasmic PGC-1 protein expression in the CUMS group was significantly decreased (*p* value < 0.05) when compared to the control group. However, the protein expressions of PGC-1 and ERR*α* in nuclei of hippocampal cells varied significantly in different groups. As shown in Figures 4(e) and 4(f), the CUMS group showed a significant decrease in the protein expressions of nuclear PGC-1 (*p* value < 0.01) and ERR*α* (*p* value < 0.01) when compared to the rats in the control group, while daily administration of CUR prevented these changes with a significant increase in the protein expressions of nuclear PGC-1 (*p* value < 0.01) and ERR*α* (*p* value < 0.01) in the CUMS + CUR group. In addition, administration of SR18292 significantly decreased the protein expressions of nuclear PGC-1 (*p* value < 0.01) and ERR*α* (*p* value < 0.05) in the CUMS + CUR + SR18292 group compared to the CUMS + CUR group.

**Figure 5:**
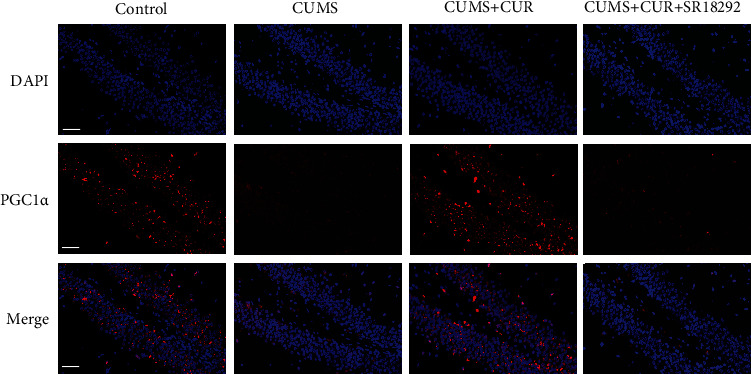
Representative images of immunofluorescence staining for PGC-1*α* in brain slices of the hippocampus. Scale bar = 50 *μ*m.

The results of the immunofluorescence staining are shown in Figure 5. Consistent with the Western blot results, CUMS rats showed a decreased PGC-1 expression when compared to the control group. The expression of PGC-1 in the CUMS + CUR group was obviously increased when compared to the CUMS group. Moreover, SR18292 significantly decreased the expression of PGC-1 in the CUMS + CUR + SR18292 group when compared to the CUMS + CUR group.

### 3.3. Effects of CUR on FNDC5 and BDNF Expressions

**Figure 6:**
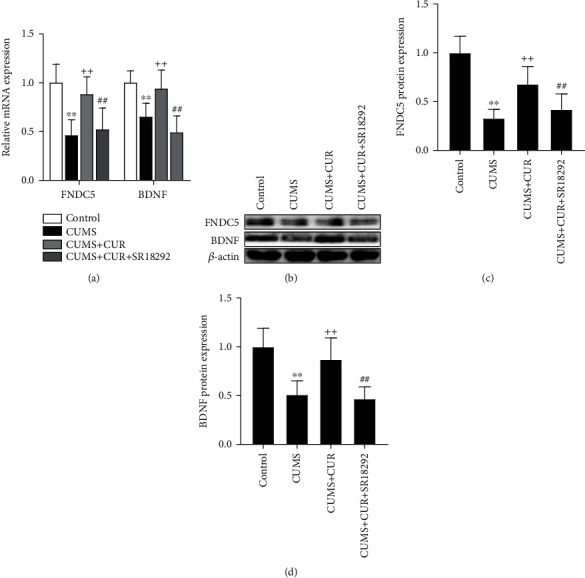
(a) Relative mRNA expressions of FNDC5 and BDNF. (b) Western blot bands indicating the protein expressions of FNDC5 and BDNF. (c) and (d) Protein expressions of FNDC5 and BDNF normalized to that of the *β*-actin internal control. Data are expressed as means ± SD (*n* = 7). ^∗^*p* < 0.05 and ^∗∗^*p* < 0.01 compared to the control group. ^+^*p* < 0.05 and ^++^*p* < 0.01 compared to the CUMS group. ^#^*p* < 0.05 and ^##^*p* < 0.01 compared to the CUMS + CUR group.

As shown in Figure 6(a), the mRNA levels of FNDC5 (*p* value < 0.01) and BDNF (*p* value < 0.01) were significantly decreased in the CUMS group compared to the control group, while the supplementation of CUR markedly increased the mRNA expressions of FNDC5 (*p* value < 0.01) and BDNF (*p* value < 0.01). Furthermore, administration of SR18292 reversed the effects of CUR and significantly reduced the mRNA expressions of FNDC5 (*p* value < 0.01) and BDNF (*p* value < 0.01).

As shown in Figures 6(b)–6(d), the Western blot results revealed that the protein expressions of FNDC5 (*p* value < 0.01) and BDNF (*p* value < 0.01) were significantly decreased in the CUMS group compared to the control group, while the supplementation of CUR markedly increased the protein expressions of FNDC5 (*p* value < 0.01) and BDNF (*p* value < 0.01). In addition, administration of SR18292 reversed the effects of CUR and significantly reduced the protein expressions of FNDC5 (*p* value < 0.01) and BDNF (*p* value < 0.01).

### 3.4. Effects of CUR on the Neuronal Proliferation and Apoptosis

**Figure 7:**
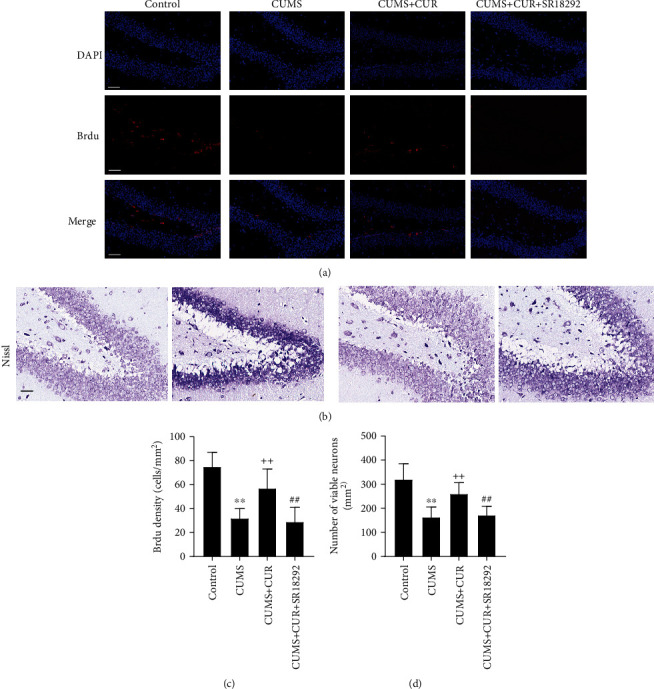
(a) Representative images of immunofluorescence staining for Brdu positive cells. (b) Representative images of Nissl-stained neurons. (c) Quantification of BrdU fluorescence in the DG area of the hippocampus. (d) Quantification of viable neurons in the DG area of the hippocampus. Scale bar = 50 *μ*m. Data are expressed as means ± SD (*n* = 7). ^∗^*p* < 0.05 and ^∗∗^*p* < 0.01 compared to the control group. ^+^*p* < 0.05 and ^++^*p* < 0.01 compared to the CUMS group. ^#^*p* < 0.05 and ^##^*p* < 0.01 compared to the CUMS + CUR group.

The immunofluorescence staining of BrdU was used to determine the neuronal proliferation in hippocampus tissue. As shown in Figures 7(a) and 7(c), the quantitative results of the immunofluorescence staining showed that CUMS significantly decreased the number of BrdU+ cells (*p* value < 0.01) in the hippocampus in comparison with the control group. Administration of CUR significantly increased the number of BrdU+ cells (*p* value < 0.01) when compared to the CUMS group. Additionally, SR18292 reversed the effects of CUR and significantly decreased the number of BrdU+ cells (*p* value < 0.01).

Nissl staining was used to identify apoptotic neurons in hippocampus tissue. As shown in Figures 7(b) and 7(d), the quantitative results of the Nissl staining showed that the number of viable neurons (*p* value < 0.01) in the CUMS group was significantly reduced compared to that in the control group. Administration of CUR significantly increased the number of viable neurons (*p* value < 0.01) when compared to the CUMS group. Moreover, SR18292 reversed the effects of CUR and significantly decreased the number of viable neurons (*p* value < 0.01).

**Figure 8:**
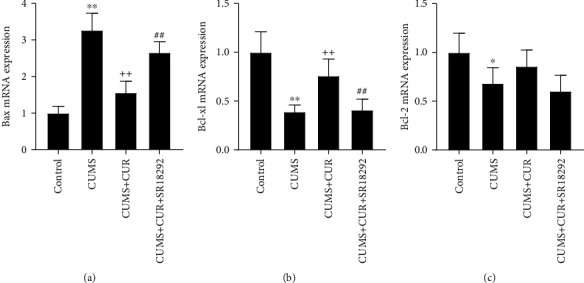
Relative mRNA expressions of (a) Bax, (b) Bcl-xl, and (c) Bcl-2. Data are expressed as means ± SD (*n* = 7). ^∗^*p* < 0.05 and ^∗∗^*p* < 0.01 compared to the control group. ^+^*p* < 0.05 and ^++^*p* < 0.01 compared to the CUMS group. ^#^*p* < 0.05 and ^##^*p* < 0.01 compared to the CUMS + CUR group.

As shown in Figures 8(a)–8(c), the CUMS group significantly increased the mRNA expression of Bax (*p* value < 0.01) and decreased the mRNA levels of Bcl-2 (*p* value < 0.01) and Bcl-xl (*p* value < 0.05), while the CUMS + CUR group showed significant decrease in the mRNA expression of Bax (*p* value < 0.01) and increase in the mRNA levels of Bcl-2 (*p* value < 0.01). Furthermore, administration of SR18292 reversed the effects of CUR and dramatically induced the mRNA expression of Bax and reduced the mRNA levels of Bcl-2 (*p* value < 0.01).

## 4. Discussion

The present study provides novel evidence supporting the link between the antidepressant-like activities of CUR and the PGC-1*α*/FNDC5/BDNF pathway. We observed that the administration of CUR normalized behavioral changes in rats exposed to CUMS, showing the antidepressant-like activities of CUR. We also found that CUR could effectively prevent CUMS-induced reduction of PGC-1*α*, ERR*α*, FNDC5, and BDNF expressions. Besides, CUR could promote cell proliferation and suppress neuronal apoptosis induced by CUMS. Additionally, we found the PGC-1*α* inhibitor, SR1829, markedly reversed the antidepressant-like effects of CUR in the behavioral tests, as well as the effects of CUR in the expressions of PGC-1*α*, ERR*α*, FNDC5, and of BDNF. These findings demonstrate that CUR may have the potential to reverse the development of depression, and they indicate that the antidepressant mechanisms of CUR are associated with the activation of the PGC-1*α*/FNDC5/BDNF pathway.

CUMS, a valid model of depression for rodents [35], was established in the present study. Consistent with previous reports [36, 37], 6 weeks of stress exposure induced the rats in the CUMS group to a depression-like state. Results of behavioral tests indicated that administration of CUR was able to attenuate the behavioral abnormalities induced by CUMS, reflecting the antidepressant-like properties of CUR [15, 16, 38]. However, the administration of PGC-1 inhibitor SR18292 reversed the beneficial effects of CUR and induced abnormal behaviors. This fact indicates that that PGC-1 might play an important role in the antidepressant-like effects of CUR.

Numerous studies have demonstrated decreased expression levels of BDNF in the brains of depressed rats [15, 16, 38]. It has been reported that CUR seems to have the ability to reverse CUMS-induced decreases in brain BDNF levels [15, 39]. Consistent with the findings of previous studies, the present study found significantly increased expression of BDNF in CUMS + CUR rats compared to CUMS rats, confirming the capacity of CUR to enhance the expression of BDNF in hippocampal neurons. It has been demonstrated that PGC-1*α* is closely related to the regulation of BDNF expression in the hippocampus through an FNDC5-dependent mechanism [10, 14, 40]. Wrann et al. [10] reported that endurance exercise could induce increased expression of BDNF through activating the PGC-1*α*/FNDC5/BDNF pathway in the hippocampus. In their study, results showed that neuronal FNDC5 gene expression is regulated by PGC-1*α*, and that FNDC5 regulates BDNF gene expression in a cell-autonomous manner. In our present study, we also examined the effects of CUR on the expressions of PGC-1*α* and FNDC5. Our results showed that the levels of PGC-1*α* and FNDC5 were highly increased in CUMS + CUR rats compared to CUMS rats. Combined with the results of behavioral tests and the increased expressions of PGC-1*α*, FNDC5, and BDNF in CUMS + CUR rats, this evidence leads to a conclusion that the antidepressant-like effects of CUR are closely linked to the positive regulation of the PGC-1*α*/FNDC5/BDNF pathway.

Furthermore, to explore whether PGC-1*α* is required for CUR's regulation of BDNF expression, the PGC-1*α* inhibitor SR1829 was used to inhibit the expression of PGC-1*α*. After administration of SR1829, we examined the expression of PGC-1*α* in the hippocampus. SR1829 effectively decreased PGC-1*α* expression in this area. The expression levels of FNDC5 and BDNF were also significantly reduced. The abnormal behaviors induced by SR1829 and the decreased expressions of PGC-1*α*, FNDC5, and BDNF implied that PGC-1*α* plays an essential role in the induction of BDNF by CUR. As a transcriptional coactivator, PGC-1*α* does not bind to the DNA itself but interacts with transcription factors to execute its effects on gene expression [41]. The orphan nuclear receptor ERR*α* is a central metabolic regulator and is known to be a very important interactor with PGC-1*α* [42, 43]. The interaction of PGC-1a with ERR*α* has been proved to be crucial for FNDC5 gene expression [10]. We therefore asked if CUR activates the transcription of PGC-1*α* and ERR*α*, thereby leading to increased FNDC5 and BDNF levels [10, 11]. Indeed, we found that CUR effectively restored the decreased gene expressions of PGC-1*α* and ERR*α* induced by CUMS. Remarkably, the results of Western blot showed significantly increased nuclear protein expressions of PGC-1*α* and ERR*α* in CUMS+CUR rats compared with the CUMS rats, while the cytoplasmic protein expressions of PGC-1*α* and ERR*α* showed no significant difference in different groups. These data demonstrate that CUR administration not only elevated the expressions of PGC-1*α* and ERR*α* but also promoted PGC-1*α* and ERR*α* nuclear translocation. In summary, the above results show that the PGC-1*α*/FNDC5/BDNF pathway was inhibited under CUMS, and administration of CUR stimulated the transcription of PGC-1*α* and ERR*α*, enhanced PGC-1*α* and ERR*α* translocation from cytoplasm to nucleus, and ultimately activated the PGC-1*α*/FNDC5/BDNF pathway.

We also investigated the effects of CUR on the proliferation and survival of neuronal cells [44]. Previous studies showed decreased neuronal proliferation and increased cell apoptosis in depressed rats [45, 46]. We observed the same results in CUMS model rats. The present study also represented the neuroprotective capacity of CUR. The immunofluorescence staining showed that CUR led to a significant increase in the average number of BrdU positive cells in the hippocampus, suggesting the potential role of CUR in promoting neuronal proliferation in rats. In addition, CUR significantly increased the number of viable neurons in the DG area of the hippocampus, markedly inhibited the gene expression of proapoptotic Bax, and enhanced the gene expression of antiapoptotic factor Bcl-xl. Taken together, these results demonstrate that CUR effectively suppressed the apoptosis induced by CUMS. As the most abundant neurotrophic factor in the brain, BDNF has been suggested to play a crucial role in the regulation of neuronal proliferation and survival [47]. Given the neurotrophic actions of BDNF and CUR's regulation of BDNF levels, we speculate that the neuroprotective capacity of CUR is due to the elevated expression of BDNF.

## 5. Conclusions

Collectively, our present study suggests that the administration of CUR can ameliorate depression-like behaviors in CUMS rats. Furthermore, our study links the activation of a metabolic regulator, PGC-1a, via FNDC5 to the increased BDNF levels induced by CUR. We provide novel evidence for the hypothesis that the antidepressant-like effects of CUR might be mediated by restoring changes in the PGC-1*α*/FNDC5/BDNF signaling pathway in the hippocampus of CUMS rats.

## Figures and Tables

**Table 1:** Primer sequences used for the qPCR analysis.

Gene	Direction	Primer sequences	Amplicon length (bp)	Accession number
PGC-1*α*	Forward Reverse	5′-GAACCATGCAAACCACACCC-3′ 5′-GGAGGGTCATCGTTTGTGGT-3′	162	NM_008904
ERR*α*	Forward Reverse	5′-AACCTGAGAAGCTGTACGCC-3′ 5′-CCATCCACACACTCTGCAGT-3′	186	NM_007953
FNDC5	Forward Reverse	5′-TATATCGTCCACGTGCAGGC-3′ 5′-ACGACGATGATCAGCACCTC-3′	179	NM_027402
BDNF	Forward Reverse	5′-TACCTGGATGCCGCAAACAT-3′ 5′-CGACATGTCCACTGCAGTCT-3′	135	NM_001048141
Bax	Forward Reverse	5′-GAACCATCATGGGCTGGACA-3′ 5′-GTGAGTGAGGCAGTGAGGAC-3′	157	NM_007527
Bcl-xl	Forward Reverse	5′-AGGCTGGCGATGAGTTTGAA-3′ 5′-AGAAGAAGGCCACAATGCGA-3′	159	NM_009743
Bcl-2	Forward Reverse	5′-GAACTGGGGGAGGATTGTGG-3′ 5′-CATCCCAGCCTCCGTTATCC-3′	164	NM_009741
*β*-Actin	Forward Reverse	5′-CCACCATGTACCCAGGCATT-3′ 5′-CGGACTCATCGTACTCCTGC-3′	189	NM_007393

PGC-1*α*: Peroxisome proliferator-activated receptor *γ* coactivator-1 alpha; ERR*α*: Estrogen-related Receptor alpha; FNDC5: Fibronectin type III Domain-Containing 5; BDNF: Brain-derived Neurotrophic Factor; Bax: Bcl2-associated X protein; Bcl-xl: Bcl2-like 1; Bcl-2: B cell leukemia/lymphoma 2.

## Data Availability

The datasets analyzed during the current study are available from the corresponding authors on reasonable request.

## References

[1] Murray C. J. L. (2018). Global, regional, and national incidence, prevalence, and years lived with disability for 354 diseases and injuries for 195 countries and territories, 1990-2017: a systematic analysis for the global burden of disease study 2017. *The Lancet*.

[2] Troubat R., Barone P., Leman S. (2021). Neuroinflammation and depression: a review. *European Journal of Neuroscience*.

[3] van Calker D., Serchov T., Normann C., Biber K. (2018). Recent insights into antidepressant therapy: distinct pathways and potential common mechanisms in the treatment of depressive syndromes. *Neuroscience & Biobehavioral Reviews*.

[4] Autry A. E., Monteggia L. M. (2012). Brain-derived neurotrophic factor and neuropsychiatric disorders. *Pharmacological Reviews*.

[5] Björkholm C., Monteggia L. M. (2016). BDNF - a key transducer of antidepressant effects. *Neuropharmacology*.

[6] Arany Z., Foo S., Ma Y. (2008). HIF-independent regulation of VEGF and angiogenesis by the transcriptional coactivator PGC-1*α*. *Nature*.

[7] Lin J., Handschin C., Spiegelman B. M. (2005). Metabolic control through the PGC-1 family of transcription coactivators. *Cell Metabolism*.

[8] Xia D. Y., Huang X., Bi C. F., Mao L. L., Peng L. J., Qian H. R. (2017). PGC-1*α* or FNDC5 is involved in modulating the effects of A*β*1-42 oligomers on suppressing the expression of BDNF, a beneficial factor for inhibiting neuronal apoptosis, A*β* deposition and cognitive decline of APP/PS1 Tg mice. *Frontiers in Aging Neuroscience*.

[9] Zhao Z., Yao M., Wei L., Ge S. (2020). Obesity caused by a high-fat diet regulates the Sirt1/PGC-1*α*/FNDC5/BDNF pathway to exacerbate isoflurane-induced postoperative cognitive dysfunction in older mice. *Nutritional Neuroscience*.

[10] Wrann C. D., White J. P., Salogiannnis J. (2013). Exercise Induces Hippocampal BDNF through a PGC-1*α*/FNDC5 Pathway. *Cell Metabolism*.

[11] Belviranlı M., Okudan N. (2018). Exercise training protects against aging-induced cognitive dysfunction via activation of the hippocampal PGC-1*α*/FNDC5/BDNF pathway. *Neuromolecular Medicine*.

[12] Wrann C. D. (2015). FNDC5/Irisin – their role in the nervous system and as a mediator for beneficial effects of exercise on the brain. *Brain Plasticity*.

[13] Young M. F., Valaris S., Wrann C. D. (2019). A role for FNDC5/Irisin in the beneficial effects of exercise on the brain and in neurodegenerative diseases. *Progress in Cardiovascular Diseases*.

[14] Jodeiri Farshbaf M., Ghaedi K., Megraw T. L. (2016). Does PGC1*α*/FNDC5/BDNF elicit the beneficial effects of exercise on neurodegenerative disorders?. *Neuromolecular Medicine*.

[15] Zhang W., Guo Y., Han W. (2019). Curcumin relieves depressive-like behaviors via inhibition of the NLRP3 inflammasome and kynurenine pathway in rats suffering from chronic unpredictable mild stress. *International Immunopharmacology*.

[16] Liao D., Lv C., Cao L. (2020). Curcumin attenuates chronic unpredictable mild stress-induced depressive-like behaviors via restoring changes in oxidative stress and the activation of Nrf2 signaling pathway in rats. *Oxidative Medicine and Cellular Longevity*.

[17] da Silva Marques J. G., Antunes F. T., da Silva Brum L. F. (2021). Adaptogenic effects of curcumin on depression induced by moderate and unpredictable chronic stress in mice. *Behavioural Brain Research*.

[18] Xu Y., Ku B. S., Yao H. Y. (2005). The effects of curcumin on depressive-like behaviors in mice. *European Journal of Pharmacology*.

[19] al-Karawi D., al Mamoori D. A., Tayyar Y. (2016). The role of curcumin administration in patients with major depressive disorder: mini meta-analysis of clinical trials. *Phytotherapy Research*.

[20] Ng Q. X., Koh S. S. H., Chan H. W., Ho C. Y. X. (2017). Clinical use of curcumin in depression: a meta-analysis. *Journal of the American Medical Directors Association*.

[21] Fusar-Poli L., Vozza L., Gabbiadini A. (2020). Curcumin for depression: a meta-analysis. *Critical Reviews in Food Science and Nutrition*.

[22] Wang Z., Zhang Q., Huang H., Liu Z. (2021). The efficacy and acceptability of curcumin for the treatment of depression or depressive symptoms: a systematic review and meta-analysis. *Journal of Affective Disorders*.

[23] Franco-Robles E., Campos-Cervantes A., Murillo-Ortiz B. O. (2014). Effects of curcumin on brain-derived neurotrophic factor levels and oxidative damage in obesity and diabetes. *Applied Physiology, Nutrition, and Metabolism*.

[24] Hurley L. L., Akinfiresoye L., Nwulia E., Kamiya A., Kulkarni A. A., Tizabi Y. (2013). Antidepressant-like effects of curcumin in WKY rat model of depression is associated with an increase in hippocampal BDNF. *Behavioural Brain Research*.

[25] Huang Z., Zhong X. M., Li Z. Y., Feng C. R., Pan A. J., Mao Q. Q. (2011). Curcumin reverses corticosterone-induced depressive-like behavior and decrease in brain BDNF levels in rats. *Neuroscience Letters*.

[26] Willner P., Towell A., Sampson D., Sophokleous S., Muscat R. (1987). Reduction of sucrose preference by chronic unpredictable mild stress, and its restoration by a tricyclic antidepressant. *Psychopharmacology*.

[27] Forbes N. F., Stewart C. A., Matthews K., Reid I. C. (1996). Chronic mild stress and sucrose consumption: validity as a model of depression. *Physiology & Behavior*.

[28] Gronli J., Murison R., Fiske E. (2005). Effects of chronic mild stress on sexual behavior, locomotor activity and consumption of sucrose and saccharine solutions. *Physiology & Behavior*.

[29] Cryan J. F., Page M. E., Lucki I. (2005). Differential behavioral effects of the antidepressants reboxetine, fluoxetine, and moclobemide in a modified forced swim test following chronic treatment. *Psychopharmacology*.

[30] Bodnoff S. R., Suranyi-Cadotte B., Aitken D. H., Quirion R., Meaney M. J. (1988). The effects of chronic antidepressant treatment in an animal model of anxiety. *Psychopharmacology*.

[31] Hirano S. (2012). Western blot analysis. *Methods in Molecular Biology*.

[32] Chen X., Pan Z., Fang Z. (2018). Omega-3 polyunsaturated fatty acid attenuates traumatic brain injury-induced neuronal apoptosis by inducing autophagy through the upregulation of SIRT1-mediated deacetylation of Beclin-1. *Journal of Neuroinflammation*.

[33] Hicks J. M. (1984). Fluorescence immunoassay. *Human Pathology*.

[34] Tang M., Zhang M., Cai H. (2016). Maternal diet of polyunsaturated fatty acid altered the cell proliferation in the dentate gyrus of hippocampus and influenced glutamatergic and serotoninergic systems of neonatal female rats. *Lipids in Health and Disease*.

[35] Willner P. (1997). Validity, reliability and utility of the chronic mild stress model of depression: a 10-year review and evaluation. *Psychopharmacology*.

[36] Tang M., Jiang P., Li H. (2015). Antidepressant-like effect of n-3 PUFAs in CUMS rats: role of tPA/PAI-1 system. *Physiology & Behavior*.

[37] Tang M., Jiang P., Li H. (2015). Fish oil supplementation alleviates depressant-like behaviors and modulates lipid profiles in rats exposed to chronic unpredictable mild stress. *BMC Complementary and Alternative Medicine*.

[38] Fan C., Song Q., Wang P. (2018). Curcumin protects against chronic stress-induced dysregulation of neuroplasticity and depression-like behaviors via suppressing IL-1*β* pathway in rats. *Neuroscience*.

[39] Zhang L., Luo J., Zhang M., Yao W., Ma X., Yu S. Y. (2014). Effects of curcumin on chronic, unpredictable, mild, stress-induced depressive-like behaviour and structural plasticity in the lateral amygdala of rats. *The International Journal of Neuropsychopharmacology*.

[40] Islam M. R., Young M. F., Wrann C. D. (2018). The role of FNDC5/Irisin in the nervous system and as a mediator for beneficial effects of exercise on the brain. *Hormones, Metabolism and the Benefits of Exercise*.

[41] Spiegelman B. M. (2007). Transcriptional control of energy Homeostasis through the PGC1 coactivators. *Novartis Foundation Symposia*.

[42] Luo J., Sladek R., Carrier J., Bader J. A., Richard D., Giguere V. (2003). Reduced fat mass in mice lacking orphan nuclear receptor estrogen-related receptor *α*. *Molecular and Cellular Biology*.

[43] Schreiber S. N., Emter R., Hock M. B. (2004). The estrogen-related receptor (ERR ) functions in PPAR coactivator 1 (PGC-1 )-induced mitochondrial biogenesis. *Proceedings of the National Academy of Sciences of the United States of America*.

[44] Pilar-Cuéllar F., Vidal R., Díaz A. (2013). Neural plasticity and proliferation in the generation of antidepressant effects: hippocampal implication. *Neural Plasticity*.

[45] Tong L., Gong Y., Wang P. (2017). Microglia loss contributes to the development of major depression induced by different types of chronic stresses. *Neurochemical Research*.

[46] Kreisel T., Frank M. G., Licht T. (2014). Dynamic microglial alterations underlie stress-induced depressive-like behavior and suppressed neurogenesis. *Molecular Psychiatry*.

[47] Di Carlo P., Punzi G., Ursini G. (2019). Brain-derived neurotrophic factor and schizophrenia. *Psychiatric Genetics*.

[48] Wu Y., Sun F., Guo Y. (2021). Curcumin relieves CUMS-induced depressive-like behaviors through PGC-1*α*/FNDC5/BDNF pathway. *Research Square*.

